# Optimization of Magnetic Field-Assisted Synthesis of Carbon Nanotubes for Sensing Applications

**DOI:** 10.3390/s141018474

**Published:** 2014-10-07

**Authors:** Grzegorz Raniszewski, Marcin Pyc, Zbigniew Kolacinski

**Affiliations:** Lodz University of Technology, Stefanowskiego Str. 18/22, Lodz 90-924, Poland; E-Mails: marcin@pyc.pl (M.P.); zbigniew.kolacinski@p.lodz.pl (Z.K.)

**Keywords:** carbon nanotubes, magnetic field, plasma

## Abstract

One of the most effective ways of synthesizing carbon nanotubes is the arc discharge method. This paper describes a system supported by a magnetic field which can be generated by an external coil. An electric arc between two electrodes is stabilized by the magnetic field following mass flux stabilization from the anode to the cathode. In this work four constructions are compared. Different configurations of cathode and coils are calculated and presented. Exemplary results are discussed. The paper describes attempts of magnetic field optimization for different configurations of electrodes.

## 1. Introduction

Nowadays carbon nanotubes (CNTs) are one of the most important materials in almost every industry. Discovered in 1991, CNTs quickly became attractive for many companies. Their unique electrical, mechanical, optical, thermal and chemical properties make them useful for many engineering applications. One of the oldest methods for carbon nanotube production is an electric arc discharge. In this method two graphite electrodes are used–one the anode as a source of carbon for carbon nanotubes formation and the other as the cathode where carbon deposits are formed. The high temperature of the electric arc discharge between graphite electrodes leads to evaporation of carbon material from the anode and subsequent deposition on the relative cold cathode surface. This deposit contains carbon nanotubes. In order to improve the efficiency of the system an external magnetic field can be applied [1,2]. The magnetic field is used to produce stable arc in the cathode region. It allows one to create an intense and uniform vapor which is generated in the anode spot. Due to the magnetic field generated by an external coil or permanent magnets, it is possible to stabilize the arc movement and in effect the cathode region temperature. The arc temperature in this region and the temperature of the plasma column appear to be crucial for the synthesis and the quality of the CNTs.

## 2. Carbon Nanotubes for Sensing Applications

Due to their small size carbon nanotubes may be used in micro- and nanodevices such as sensors. Large surface area, high aspect ratio, unique electrical and thermal properties make the carbon nanotubes systems more precise and more sensitive than macro-sized devices. In the area of sensors an infrared (IR) light detector with higher sensitivity than existing technology has been demonstrated. The device, known as an IR bolometer, is made by suspending a 0.5 mm wide layer of single-walled CNTs over a 3.5 mm gap between two electrical contacts. This layer is heated by incident IR radiation, causing its resistance to change. It is also possible to use CNTs as a gas detector because of the existing interactions between nanotubes and gas molecules. The gas can be ionized at voltages that are up to 65% lower than in traditional sensors [3].

Chemical and gas sensors sense simple molecules containing only a few atoms. Chemical sensors based on carbon nanotubes detect chemicals such as NO_2_ and NH_3_. Conventional sensors for NO_2_ and NH_3_ usually work over 550 K and provide limited sensitivity. Semiconducting gas sensors use conductance changes between the semiconducting single-walled carbon nanotubes and gas molecules. These sensors operate at room temperature and can detect several parts per trillion of measured contaminants [4]. Nanotubes can be used to measure ion concentrations and types. In modified electrolyte-insulator-semiconductors carbon nanotubes can be placed on the dielectric. When a large amount of ions are present, an electric field occurs across the insulator. This field reduces the energy gap of the semiconducting nanotube what leads to a source-drain conductance increase [5]. Dielectric gas sensors measure dielectric constant changes when a layer of CNTs is exposed to different gas molecules [6]. Absorption-based gas sensors use a cantilever covered by single- or multiwalled carbon nanotubes. The gas molecules combine with the carbon nanotubes what leads to change of the cantilever mass, and accordingly to a change in its oscillating frequency [7,8]. Carbon nanotubes can be also used in biological applications. In electrochemical catalytic amplifiers CNTs work as catalysts, as catalysts and DNA anchors, and as catalysts and enzyme anchors [9–11]. Another area of biological application are nanooptical transducers. In these sensors enzymes, *i.e.*, glucose oxidase, and carbon nanotubes are placed in the solution. Laser light is used to illuminate the solution and the fluorescence of single walled nanotubes is detected [12].

Carbon nanotubes are thus very promising products for sensing applications. In practice, the market potential depends on technical and manufacturing feasibility. Thus, identifying methods to produce the desired, product repeatably is very important. Apart from the CVD method, which seems to be very suitable for this, one solution is to improve the electric arc discharge methods. The proposed improvement involves the use of an additional external magnetic field in the system.

## 3. Experimental Section

**Figure 1.**
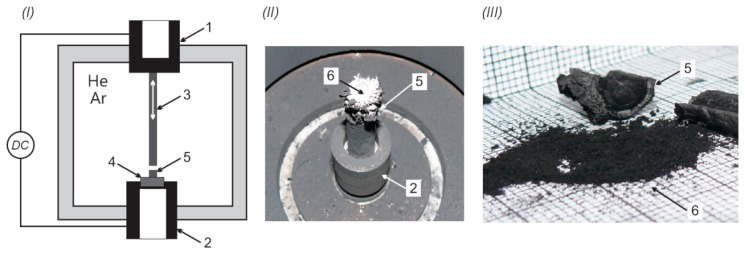
The scheme of the system for carbon nanotubes synthesis (**I**); cathode deposit during the process (**II**) and separated soft core from cathode deposit (**III**) where: (1) water cooled anode holder; (2) water cooled cathode holder; (3) graphite anode; (4) cathode; (5) cathode deposit; (6) core with CNTs.

The main disadvantage of arc methods is the final product quality—the deposit growth rate and carbon nanotube purity. These parameters depend on the stability of the arc. Electric arc movement impacts the temperature, and thus in effect the carbon elements' flux. Irregular distribution of the carbon vapor stream reduces the efficiency and flow symmetry of molecules which may form carbon nanotubes. In our system the arc discharge burns between two graphite electrodes in a steel reaction chamber containing helium at a pressure in a range from 0.2 to 0.6 bar. The temperature of the discharge leads to anode evaporation and decomposition on a cooled cathode surface as a cathode deposit. The deposit consists of two main parts—a soft core containing CNTs and an external hard shell. Figure 1 presents the system and examples of deposits.

The circuit parameters, the distance between the electrodes and the pressure were adjusted automatically by the PLC so that the optimal arc temperature on the cathode surface was obtained.

Based on authors' experience [13–15] and on the literature [16–20] the following parameters values are required:

- the arc current should be between 170 and 300 A/cm^2^ (approximately 50–80 A),
- the anode diameter should be lower than 10 mm,
- the distance between electrodes should be 0.5–5 mm.

Usually the distance does not exceed 3 mm due to efficiency drop over the 3 mm distance. Arc voltage is usually within the range of 15 to 25 V, but for carbon electrodes without introduced catalyst this value will be confined to about 21–22 V. The arc burns in helium under low pressure but argon or mixture of argon and helium can also be used.

## 4. Results and Discussion

In literature, we can find many solutions for plasma column stabilization by an external magnetic field [21–26]. Due to the complex shape of the anode and the cathode, the Finite Element Method (FEM) was chosen for the analysis. FEM is used to obtain solutions to partial differential or integral equations that cannot or are difficult to solve by analytic methods. In order to optimize the magnetic field distribution, a 2d-FEM model of the plasma column was built and a series of simulations of the magnetic field for different cathode and cathode holder geometries were performed. In order to obtain precise results the model was meshed with 160,000 quadrilateral elements and solved in terms of vector potential and equipotential line contours. Options with the anode holder and cathode holder made of magnetic and non-magnetic material were considered.

**Figure 2.**
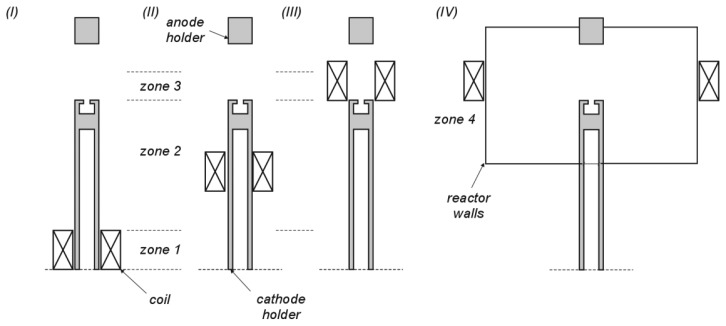
Diagram of the coils placed in different locations (**I**–**IV**) where (1) hollowed, water-cooled zone; (2) central zone; (3) top zone; (4) zone outside the chamber.

According to the experimental system and technical limitations used, it was assumed that the induction coil is placed on the hollowed and water-cooled cathode. A schematic diagram is shown in Figure 2. The cathode holder is divided into three zones: (I) bottom, water-cooled zone; (II) central zone; (III) top zone (area of carbon nanotubes deposition). Additional zone (IV) refers to coils placed outside the research chamber.

It can be found in the literature that systems which employ coils placed at a cathode deposit level are more common. Because of the high temperature of the plasma column (as high as 5000 K in the axis) between anode and cathode solutions with coils close to the arc (case III in Figure 2) are only a theoretical. Case IV with coils outside the chamber is limited by the experimental set-up geometry.

**Figure 3.**
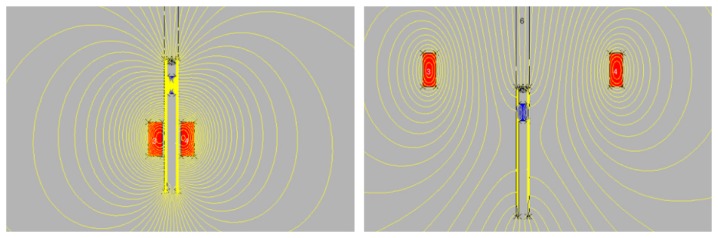
Distribution of magnetic field lines. Coil current = 5 A, 390 turns. Cathode holder made of steel.

Then the calculations for ferromagnetic cathode holder and coil placed on it were made. Figure 3 shows distribution of magnetic lines in two cases–coils on the holder in the central zone (II) and coils outside the chamber (IV).

**Figure 4.**
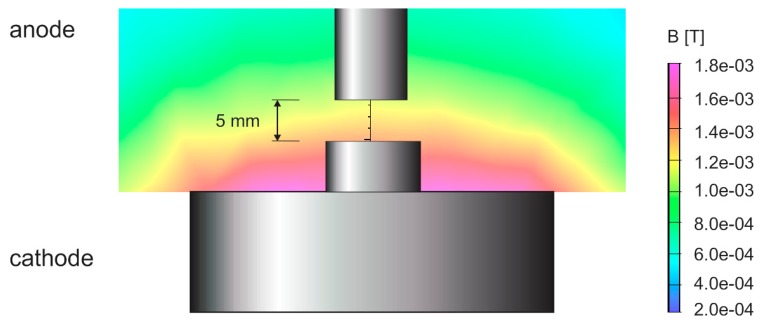
Magnetic field distribution over the cathode surface.

**Figure 5.**
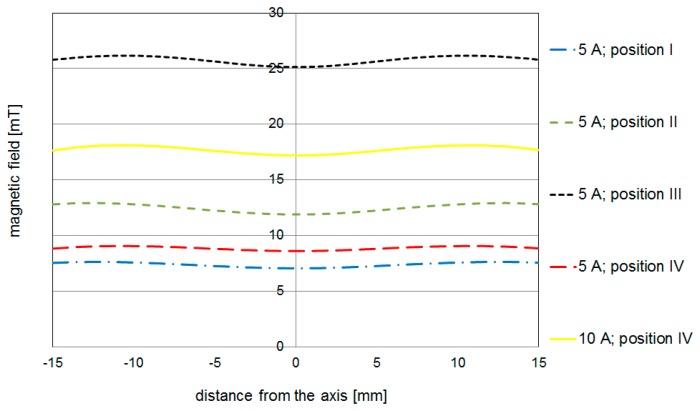
Magnetic field strength distribution on the cathode surface for coil placed in different zones (390 coils, 5 and 10 A).

The distribution of magnetic lines in the area close to the cathode surface is very similar. In Figure 4, the magnetic force for 390 turns and 5 A placed on the bottom of cathode holder is shown. Figure 5 compares the magnetic field strength on the surface of the cathode for different coil placements. It can be noticed that in the system where the coil is outside the chamber (about 20 cm from the axis) the magnetic field distribution is almost the same.

If various cases of coil position are compared it can be seen that the use of the systems with the coil in a 20 cm distance from the arc (outside the reaction chamber) and the coil placed in the region of the cathode holder give similar results (the same range of the magnetic field intensity).

**Figure 6.**
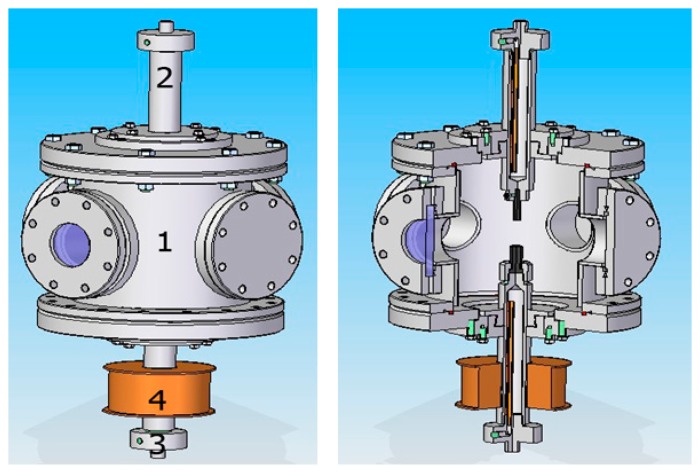
Modified research set-up where (1) main chamber; (2) anode holder; (3) cathode holder; (4) electromagnetic coil.

Based on simulations the arc discharge carbon nanotubes synthesis system has been modified by applying the magnetic field (Figure 6). The field is produced by applying a DC-powered electromagnet positioned axially on the cathode holder.

In order to improve the carbon nanotube production efficiency an external magnetic field is generated by a coil on the cathode holder. A stable arc allows the creation of a uniform vapor stream from the anode to the cathode and also to stabilize carbon molecules flux which results in a mass flow efficiency increase. The magnetic field may also be generated by permanent magnets located inside the reaction chamber but the then temperature in the chamber would reduce the lifetime of these magnets.

To determine the relation between the CNT growth according to the electrical factors, a variety of parameters such as the electric arc current and the voltage, the pressure inside the chamber and the solenoid current were recorded during the tests.

**Figure 7.**
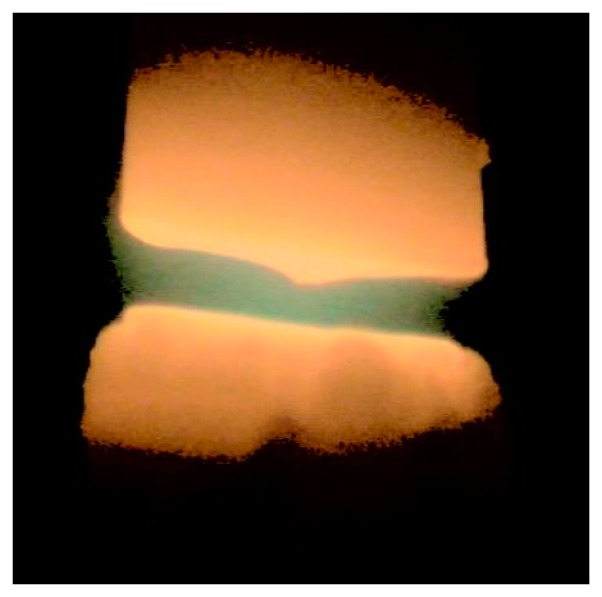
Plasma column supported by external magnetic field (unnatural colors).

Carbon decomposition occurred in the arc discharge between two graphite electrodes in a steel reaction chamber containing helium under reduced pressure. The gas introduced into the chamber did not react with the carbon electrode material. The temperature in the discharge led to the formation of the cathode deposit, transferring the material directly from the graphite anode. The stabilization of the arc by the presence of the magnetic field made it possible to create an intense and steady stream of vapor transferred from the anode to the cathode in order to ensure the effective mass flow of material. Figure 7 shows an arc column supported by a magnetic field.

Diagnosis of the carbon arc plasma was carried out using optical spectroscopy. The research showed that the arc column temperature measured in its axis is in the 4800–5600 K range, and is dependent on the length of the arc column. The temperature distribution also differs in cases with and without an external magnetic field. The magnetic field stabilizes arc movement and consequently stabilizes temperature distribution, which in turn affects the carbon decomposition. The carbon nanotube synthesis depends on the ratio between small carbon particles and large multi-atom carbons.

Comparing the distribution of carbon vapors and the carbon deposits formed under similar conditions (current, pressure, voltage, temperature) no major differences in the structure and composition of the cathode deposit were observed. However, the application of the magnetic field accelerates the deposit growth rate which increases the efficiency with no deterioration in the quality of the final product. In addition, cathode deposits produced with the magnetic field were characterized by more accurate symmetry in comparison to deposits which were formed without this field. It was easier to separate the soft core mechanically, which resulted not only in a more accurate cleaning of a hard outer shell of the part containing the nanotubes, but also increased the productivity. The quality of the synthesized CNT's is independent of the coil placement. Using the coil placed on a holder considerably simplifies the system, which is essential from an economical point of view. Application of a magnetic field improves deposit growth rate and deposit symmetry but—what is characteristic for arc plasma systems—the resulting CNT's still need purification [27].

## 5. Conclusions

The final product of the synthesis is a mixture of carbon nanotubes, amorphous carbon, soot, graphite, *etc.* The final composition depends on the process conditions. Therefore, purification and characterization of the final product for analysis is necessary. The research carried out in the system showed a significant role of the distance between the electrodes, which influences the diameter and the purity of the synthesized carbon nanotubes. It was stated that the increase of the distance between the electrodes results in a higher sample purity. A linear relationship between the electrode gap and the arc voltage was expected, but it appeared that the arc voltage change is proportional to the distance between the electrodes only for large distances. It was also noticed that an additional magnetic field in the system improves the quality of the deposits and accelerates the anode conversion into deposits. Appropriately arranged magnetic field lines improve the arc movement and the synthesis process. The magnetic field increases the deposit growth rate and the purity of the product. It has also been noticed that simulations of new constructions are efficient for the process of optimization. Finally, the electromagnetic field distribution modeling can be applied for the improvement of carbon nanotube quality.
